# The pharmacokinetic parameters and the effect of a single and repeated doses of memantine on gastric myoelectric activity in experimental pigs

**DOI:** 10.1371/journal.pone.0227781

**Published:** 2020-01-24

**Authors:** Jan Bures, Jaroslav Kvetina, Vera Radochova, Ilja Tacheci, Eva Peterova, David Herman, Rafael Dolezal, Marcela Kopacova, Stanislav Rejchrt, Tomas Douda, Vit Sestak, Ladislav Douda, Jana Zdarova Karasova

**Affiliations:** 1 2nd Department of Internal Medicine—Gastroenterology, Charles University, Faculty of Medicine in Hradec Kralove and University Hospital, Hradec Kralove, Czech Republic; 2 Animal Laboratory, University of Defence, Faculty of Military Health Sciences, Hradec Kralove, Czech Republic; 3 Department of Toxicology and Military Pharmacy, University of Defence, Faculty of Military Health Sciences, Hradec Kralove, Czech Republic; 4 Centre of Biomedical Research, University Hospital, Hradec Kralove, Czech Republic; 5 Institute of Clinical Biochemistry and Diagnostics, Charles University, Faculty of Medicine in Hradec Kralove and University Hospital, Hradec Kralove, Czech Republic; Weizmann Institute of Science, ISRAEL

## Abstract

**Background:**

Memantine, currently available for the treatment of Alzheimer's disease, is an uncompetitive antagonist of the N-methyl-D-aspartate type of glutamate receptors. Under normal physiologic conditions, these unstimulated receptor ion channels are blocked by magnesium ions, which are displaced after agonist-induced depolarization. In humans, memantine administration is associated with different gastrointestinal dysmotility side effects (vomiting, diarrhoea, constipation, motor-mediated abdominal pain), thus limiting its clinical use. Mechanism of these motility disorders has not been clarified yet. Pigs can be used in various preclinical experiments due to their relatively very similar gastrointestinal functions compared to humans. The aim of this study was to evaluate the impact of a single and repeated doses of memantine on porcine gastric myoelectric activity evaluated by means of electrogastrography (EGG).

**Methods:**

Six adult female experimental pigs (*Sus scrofa f*. *domestica*, mean weight 41.7±5.0 kg) entered the study for two times. The first EGG was recorded after a single intragastric dose of memantine (20 mg). In the second part, EGG was accomplished after 7-day intragastric administration (20 mg per day). All EGG recordings were performed under general anaesthesia. Basal (15 minutes) and study recordings (120 minutes) were accomplished using an EGG stand (MMS, Enschede, the Netherlands). Running spectral analysis based on Fourier transform was used. Results were expressed as dominant frequency of gastric slow waves (DF) and power analysis (areas of amplitudes).

**Results:**

Single dose of memantine significantly increased DF, from basic values (1.65±1.05 cycles per min.) to 2.86 cpm after 30 min. (p = 0.008), lasting till 75 min. (p = 0.014). Basal power (median 452; inter-quartile range 280–1312 μV^2) raised after 15 min. (median 827; IQR 224–2769; p = 0.386; NS), lasting next 30 min. Repetitively administrated memantine caused important gastric arrhythmia. Basal DF after single and repeated administration was not different, however, a DF increase in the second part was more prominent (up to 3.18±2.16 after 15 and 30 min., p<0.001). In comparison with a single dose, basal power was significantly higher after repetitively administrated memantine (median 3940; IQR 695–15023 μV^2; p<0.001). Next dose of 20 mg memantine in the second part induced a prominent drop of power after 15 min. (median 541; IQR 328–2280 μV^2; p<0.001), lasting till 120 min. (p<0.001).

**Conclusions:**

Both single and repeated doses of memantine increased DF. Severe gastric arrhythmia and long-lasting low power after repeated administration might explain possible gastric dysmotility side effects in the chronic use of memantine.

## Introduction

Memantine, an uncompetitive antagonist of the N-methyl-D-aspartate type glutamate receptors (briefly NMDA receptors), is used to treat Alzheimer's disease (AD) in humans. Under physiologic conditions, this receptor is blocked by magnesium ion, which is displaced upon agonist-induced depolarization. In humans, memantine therapy is associated with gastrointestinal dysmotility (vomiting, diarrhoea, constipation, motor-mediated abdominal pain), thus limiting its clinical use [1–3]. The exact mechanism of these side effects has not been clarified.

Pigs can be used in various preclinical experiments–as omnivores–due to their human-like gastrointestinal physiology [4–8]. Results of experimental studies help to understand mechanisms of drug side effects and this knowledge can be employed into clinical practice. Therefore, the aim of this study was to evaluate the impact of a single and repeated doses of memantine on porcine gastric myoelectric activity by means of electrogastrography (EGG), a non-invasive method for the evaluation of myoelectric activity of the stomach [9–11].

## Material and methods

### Animals

Six adult female experimental pigs (*Sus scrofa f*. *domestica*, hybrids of Czech White and Landrace breeds; 4-months old, mean weight 41.7±5.0 kg) entered the study twice.

All animals were fed with standard assorted A1 food (Ryhos, Novy Rychnov, Czech Republic) of equal amounts twice a day and had free access to drinking water. The acclimatization period was two weeks before the experiment.

### Design of the study

All EGG recordings were performed under general anaesthesia in the morning after 24 hours of fasting. Intramuscular injections of ketamine (20 mg per kg; Narkamon, Spofa, Praha, Czech Republic) and azaperone (2.2 mg per kg; Stresnil, Janssen Animal Health, Saunderton, UK) were used as an introduction to anaesthesia in all animals. Intravenous infusion of propofol (AstraZeneca AB, Stockholm, Sweden) was used for subsequent maintenance of general anaesthesia. Two vital signs were monitored, heart rate and pulse oximetry were used to secure the experiments and to assess possible myocardial effect of memantine. Memantine was administrated intragastrically by means of a flexible video-gastroscope (GIF-Q130, Olympus Optical Co, Tokyo, Japan), dedicated for animal use only. All endoscopies were video-recorded.

### Absorption and distribution of memantine

Concentrations of memantine measured in pig plasma collected after a single intragastric dose (20 mg; day 1), after a 7-day intragastric administration (20 mg per day; 20 mg; day 8) and after 7-day intragastric administration (20 mg per day) with a 40 mg intragastric extra dose three hours later on the last day (60 mg; day 8). Control and additional blood samples were drawn into heparinized tubes from the venous catheter at various time intervals up to 3 hours following the memantine intragastric administration. The blood samples (1 mL whole blood/time interval) at regular time intervals: 0, 10, 20, 30, 45, 60, 90, 120, 150 and 180 min was drawn. Plasma was prepared by centrifugation (1050 g, 10 min, 25°C, Centrifuge Z 206 A, Hermle, Germany) immediately after sampling and frozen at -80°C prior to analysis.

During the organ distribution study, the concentrations of memantine in pig plasma and selected organs or body fluids were collected after a 7-day intragastric administration of 20 mg per day, with a 40 mg extra dose on day 8 (administered 150 minutes after the last 20-mg dose). Ninety minutes after this extra dose of 40 mg of memantine, the pigs were sacrificed by means of pharmacological euthanasia (T61, Intervet International BV, Boxmeer, the Netherlands; dose of 2 mL/kg), exsanguinated and immediate autopsy was performed. The blood and selected organs (heart, lung, muscle, fat, spleen, liver, kidney, gastric and intestinal wall, selected parts of brain) were taken. For the elimination study the urine directly from bladder and bile from gallbladder were taken.

### HPLC analysis of memantine

#### Reagents and chemicals

Methanol, formic acid (both for mass spectrometry gradient grade), sodium hydroxide, diethyl ether, chloroform (all HPLC grade), memantine and amantadine (internal standard, IS) were purchased from Sigma-Aldrich (St. Louis, MO, USA). Ultrapure water was produced by Aqua Osmotic 06 (Aqua Osmotic, Tisnov, Czech Republic). Supelco Supelclean LC-WCX (1 mL, 100 mg) SPE cartridges were obtained from Sigma-Aldrich (St. Louis, MO, USA).

#### Standard solution, calibration standard

Stock solutions of memantine and amantadine (internal standard; IS, 100 μg.mL-1) were prepared in ultrapure water. Standard working solutions for calibration curves were prepared by diluting the stock solutions with water. Calibration standards were prepared by spiking blank matrix (990 μL) with 10 μL of a standard working solution to give concentrations in matrix from 2 to 10000 ng.mL-1. The IS working solution (1 μg.mL-1) was obtained by dilution of stock solution.

#### Sample preparation

Plasma, urine or bile (1 mL) was spiked with 20 μL of IS working solution. After spiking the mixture was loaded onto a SPE Supelclean LC-WCX cartridge, preconditioned with methanol (1 mL) and water (1 mL). The cartridge was washed with water (1 mL) and eluted with 5% formic acid in methanol (1 mL). Eluate was evaporated by using rotational vacuum concentrators UNIVAPO 100 ECH (UniEquip GmbH., Germany). The residue was reconstituted with 500 μL water. Finally, 25 μL were injected to LC–MS system.

Tissues samples were mixed with water in a ratio of 1:4 and subsequently homogenized at 15000 rpm for 1 min by Turax T25 (IKA Labortechnik, Staufen, Germany) and for 30 sec by ultrasonic homogenizer UP 50H (Hielscher Ultrasonics, Teltow, Germany), the mixture was centrifuged (10 min, 4°C, 5000 × g). Extraction of memantine and amantadine from tissue samples was a modification of the method published previously [12]. Supernatant (500 μL) was mixed with 150 μL of 0.05 M NaOH and 20 μL of IS working solution. The mixture was then shaken with 3 mL of diethyl ether-chloroform (7:3, v/v) for 5 min at 1500 RPM. After centrifugation (5 min, 16000 × g), the upper organic layer was transferred to a clean tube and evaporated by Vacuum Concentrator at 45°C (UNIVAPO 100 ECH, UniEquip, Planegg, Germany). The residue was reconstituted with 500 μL water. Finally, 25 μL were injected to LC–MS system.

#### LC-MS/MS equipment and parameters

LC-MS analysis was carried out using a Finnigan Surveyor plus system (Thermo Scientific, San Jose, CA, USA), consisting of a quaternary MS pump with integrated degasser and an auto sampler with integrated column oven. The chromatographic separation was performed on a Kinetex C18 column (5 μm, 50 mm × 4.6 mm I.D.) preceded by a C18 security guard cartridge (4.0 mm × 3.0 mm I.D.) both by Phenomenex (Torrance, CA, USA). Separation was attained using an isocratic elution with a flow rate of 0.5 mL.min-1. The column and tray temperature were set at 25 and 10°C, respectively. The mobile phase consisted of 0.5% formic acid in water and methanol (45:55 v/v). The run time of analysis was 3 min.

Mass spectrometry was performed on an LTQ XL linear ion trap instrument (Thermo Scientific, San Jose, CA, USA), coupled with heated electrospray ionization (HESI-II) probe operated in positive mode. After optimization, the parameters in the source were set as follows: source voltage 4.5 kV, source heater temperature 250°C, sheath gas flow 30 arb and auxiliary gas flow 10 arb. The mass spectrometer was operated in selected reaction monitoring (SRM) mode. The fragment ions *m/z* 180 → 163 and *m/z* 152 → 135 were used for quantification of memantine and amantadine, respectively. Thermo Fisher Xcalibur software was used for the analysis.

### Electrogastrography

The first EGG was recorded after a single intragastric dose of memantine (20 mg), followed by a 7-day wash-out period. The second EGG was taken after 7-day intragastric administration (20 mg per day). All EGG recordings were performed under general anaesthesia, using our own method [4,13–16]. Briefly, all animals were lying in a right lateral position. The epigastric area was shaved first and the skin was gently sandpapered afterwards. Six active self-adhesive electrodes were placed on the upper part of the abdomen, the 7th electrode (basal) was placed left of the middle sternum. A special abdominal belt (respiratory sensor) was used to identify possible artefacts due to breathing and body movements. Basal (15 minutes) and study recordings (120 minutes) were accomplished using an EGG stand (MMS, Enschede, the Netherlands). MMS software (version 8.19) was used to assess EGG recordings. Running spectral analysis (based on Fourier transform) was used for the elemental evaluation of the EGG. The results were expressed as dominant frequency of slow waves at all intervals of EGG recordings. EGG power analysis (areas of amplitudes) was accomplished afterwards in all animals.

### Statistical analysis

Data were statistically treated by means of descriptive statistics, non-paired t-test, Mann-Whitney rank sum test and Pearson product moment correlation using the SigmaStat software (Version 3.1, Jandel Corp, Erkrath, Germany).

### Ethics

The study was conducted in accordance with the Basic & Clinical Pharmacology & Toxicology policy for experimental and clinical studies [17]. The Project was approved by the Institutional Review Board of the Animal Care Committee of the University of Defence (No. 19/19), Faculty of Military Health Services, Hradec Kralove, Czech Republic. Animals were held and treated in accordance with the European Convention for the Protection of Vertebrate Animals [18].

## Results

### Absorption and distribution of memantine

**Fig 1:**
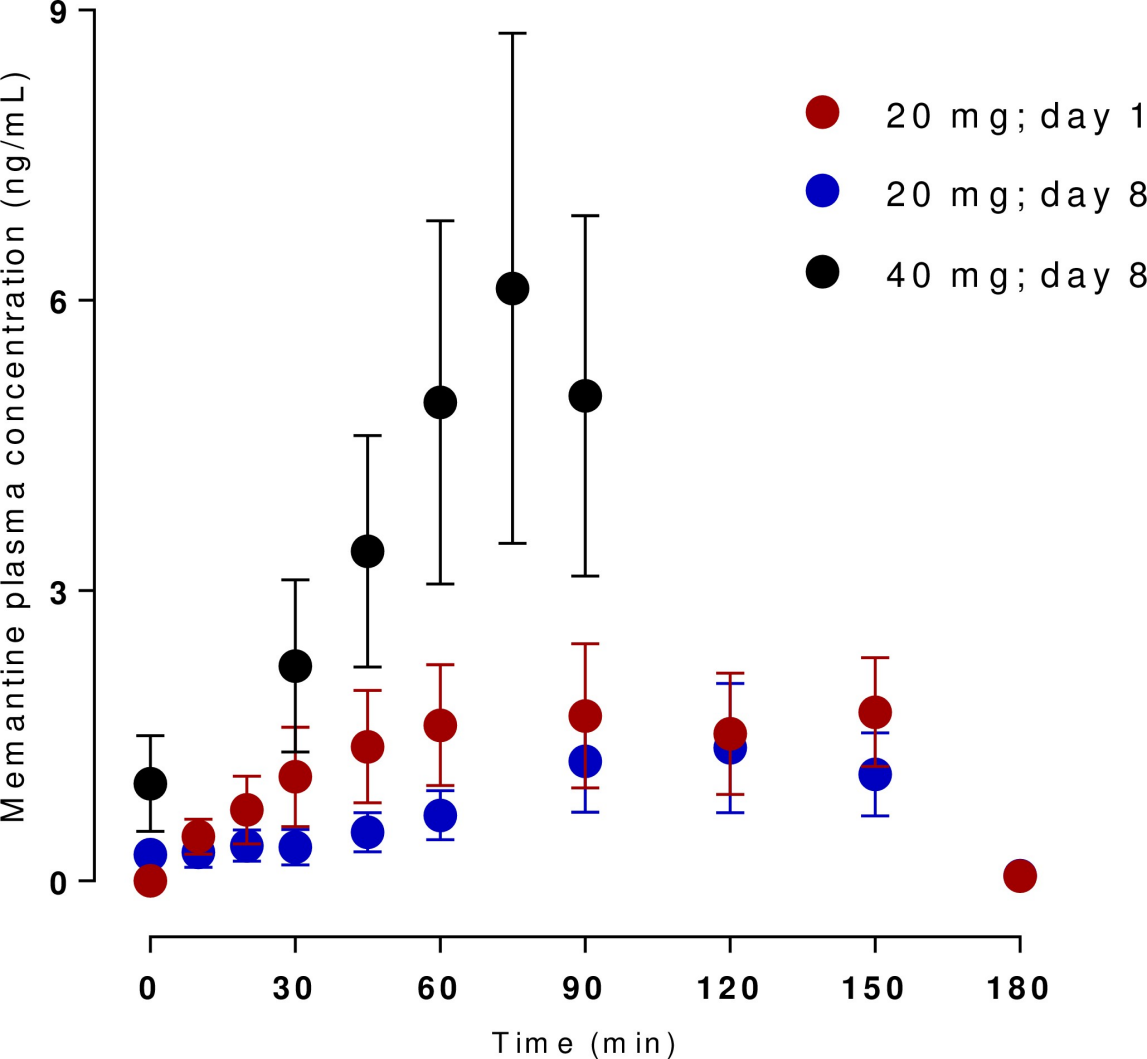
Levels of memantine measured in pig plasma collected after a single intragastric dose, after a 7-day intragastric administration and after 7-day intragastric administration with a 40 mg intragastric extra dose.

Fig 1 shows the time-concentration curves of memantine in plasma after intragastric administration in pigs *(n = 6)*.

There is little difference in concentrations after single and repeated administration after 150 minutes, but the eventual addition of 40 mg of intragastric memantine is marked by a steep rise in plasmatic concentration. The peak concentration after the last administration comes at 75 minutes and appears to stay stable until the end of the experiment.

Table 1 gives data on organ distribution after a 7-day intragastric administration (20 mg per day), with a 40 mg extra dose last day (three hours after the last 20 mg dose). To compare the bio-distributive relationships of memantine in particular tissues we also present organ/plasma ratios. The substantial difference in gastrointestinal tract to blood is probably due to the short period between last administration and the autopsy.

**Table 1:** Concentrations of memantine in pig plasma and selected organs/body fluids collected after a 7-day intragastric administration of 20 mg per day, with a 40 mg extra dose last day (administered 150 minutes after the last 20-mg dose on day 8).

Tissue	Mean ± SEM *(ng/mL or g)*	Tissue or body fluid/ plasma ratio
**plasma**	**5.08 ± 0.99**	**1.00**
stomach	433.90 ± 160.61	85.41
proximal jejunum	3066.44 ± 715.63	603.63
med jejunum	2784.51 ± 925.09	548.13
distal jejunum	731.32 ± 445.02	143.96
muscle	33.99 ± 17.28	6.69
fat	23.37 ± 3.62	4.60
spleen	68.51 ± 13.96	13.49
liver	535.88 ± 138.03	105.49
kidney	41.96 ± 8.32	8.26
heart	28.93 ± 11.33	5.69
lung	444.73 ± 116.04	87.55
medulla	49.57 ± 19.53	9.76
medulla oblongata	22.90 ± 8.94	4.51
hemisphere	15.76 ± 6.56	3.10
front lobe	26.53 ± 7.62	5.22
cerebellum	14.76 ± 5.34	2.91
pituitary gland	100.40 ± 34.70	19.76
hippocampus	52.75 ± 16.74	10.38
diencephalon	26.30 ± 7.71	5.18
urine	75.46 ± 22.75	14.85
bile	40.27 ± 12.01	7.93

### Electrogastrography

**Fig 2:**
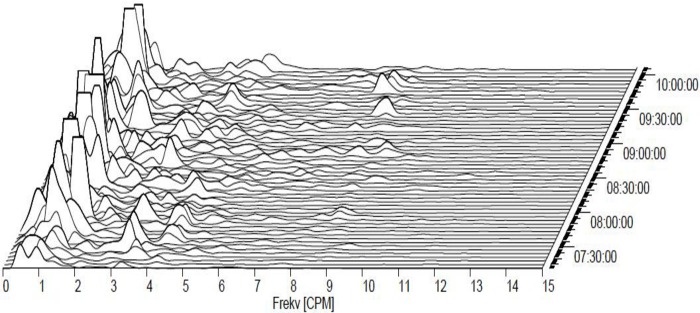
Electrogastrography. A typical pattern of combined bradygastria and gastric arrythmia in an experimental pig after a 7-day intragastric administration of memantine (20 mg per day).

A single dose of memantine nearly doubled the dominant frequency (DF), from initial values (1.65±1.05 cycles per min.) to 2.86 cpm after 30 min. (p = 0.008). This effect lasted up to 75 min. (p = 0.014). Repeated administration of memantine caused marked gastric arrhythmia (Fig 2).

**Fig 3:**
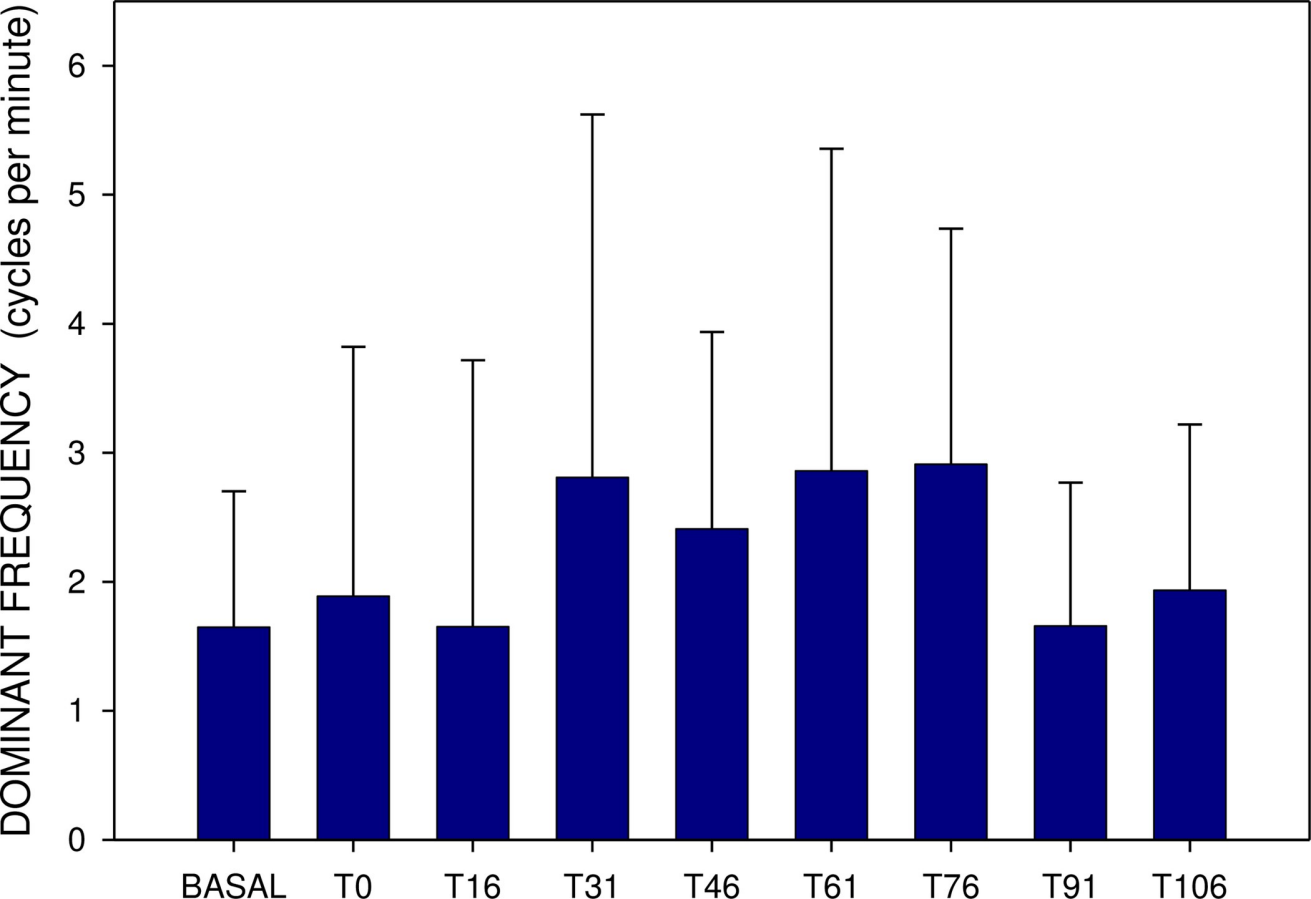
Electrogastrography. Dominant frequency (mean ± S.D.). BASAL: a 15-minute recording before the administration of a single dose of 20 mg memantine; T0: a 15-minute recording between time 0 and 15 minutes (after the administration of a single dose of 20 mg memantine) … T106: a 15-minute recording between time 106 and 120 minutes (after the administration of a single dose of 20 mg memantine).

**Fig 4:**
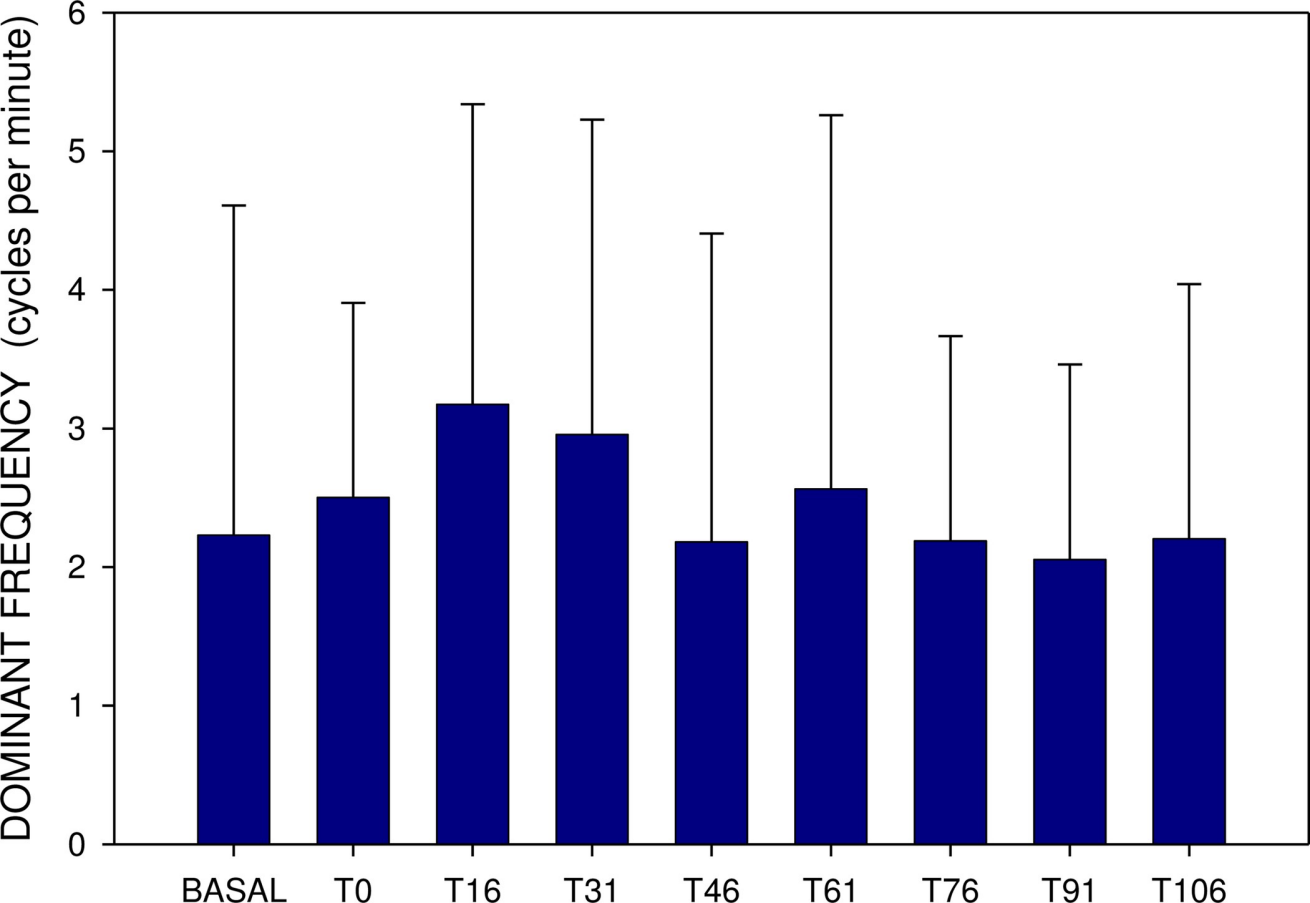
Electrogastrography. Dominant frequency (mean ± S.D.) before (BASAL) and after the administration of 20 mg memantine on day 8 (eight 15-minute intervals).

Basal DF after single and repeated administration were comparable, however, the DF increase was more prominent in the latter (up to 3.18±2.16 after 15 and 30 min., p<0.001) (Figs 3 and 4).

**Fig 5:**
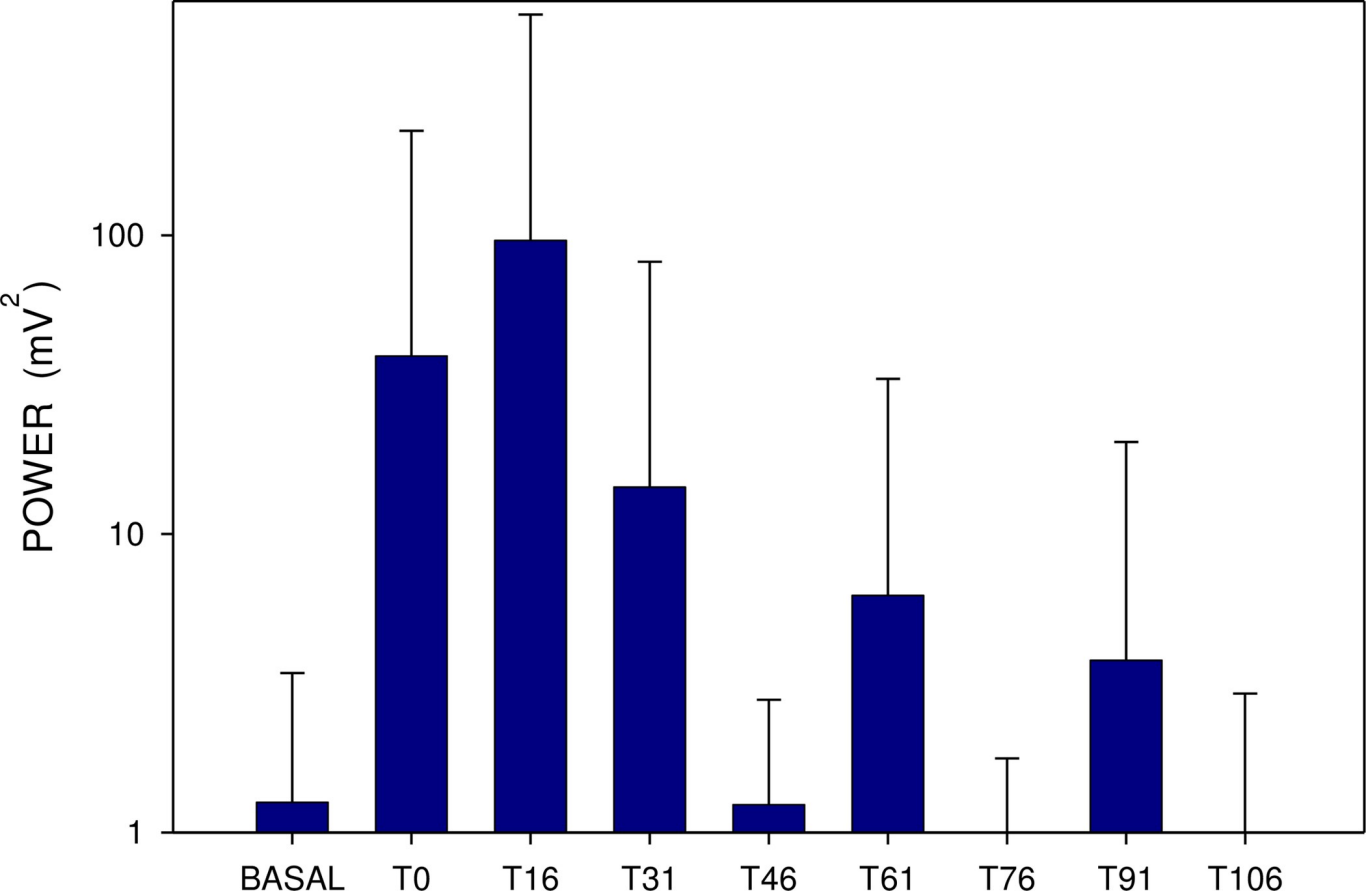
Electrogastrography. Power analysis (areas of amplitudes; mean ± S.D.) before (BASAL) and after a single dose of 20 mg memantine (eight 15-minute intervals).

**Fig 6:**
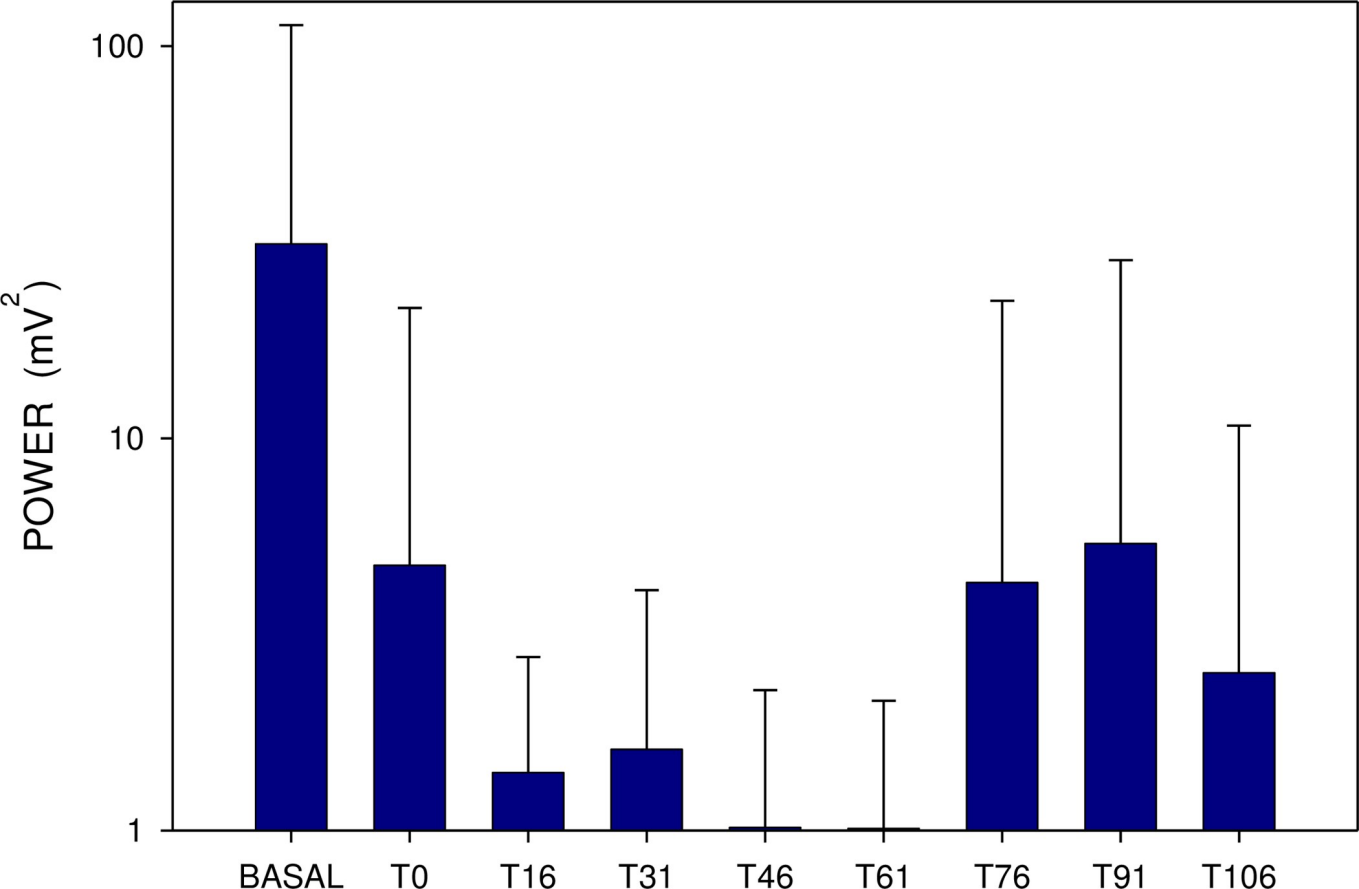
Electrogastrography. Power analysis (areas of amplitudes; mean ± S.D.) before (BASAL) and after the administration of 20 mg memantine on day 8 (eight 15-minute intervals).

Basal power (median 452; IQR 280–1312 μV^2^) also rose 15 minutes after a single dose (median 827; IQR 224–2769; p = 0.386; NS), remaining increased up to 30 min (Figs 4 and 5). In comparison, basal power was significantly higher after repetitively administered memantine (median 3940; IQR 695–15023 μV^2^; p<0.001). The additional dose of 20 mg memantine induced a prominent drop of power after 15 min (median 541; IQR 328–2280 μV^2^; p<0.001), lasting up to 120 min. (p<0.001) (Figs 5 and 6).

## Discussion

Our current study brings an important new insight into understanding the effect of memantine on gastric myoelectric activity. We used surface electrogastrography (EGG), a non-invasive method for assessment of gastric myoelectrical activity [9–11], which gives comparable results in pigs and in healthy humans, both in dominant frequency and EGG power (areas of amplitudes) [13–16]. In our current study, single dose of memantine significantly increased DF, while repetitively administrated memantine caused important gastric arrhythmia. Basal DF after single and repeated administration was not different, however, a DF increase in the second part was more prominent. In comparison with a single dose, basal power was significantly higher after repetitively administrated memantine. Next dose of 20 mg memantine in the second part induced a prominent drop of the EGG power. In our previous studies, porcine dominant frequency on EGG was fully comparable with the values of healthy humans [13–16]. We tested different types of anaesthesia previously and found no significant difference [15]. As a new finding, we currently found bradygastria within the baseline EGG recording of fasting animals. It might be, at least partially, explained by an effect of propofol. We have been using propofol recently as it shortens the general anaesthesia and makes the rapid recovery possible. Propofol can influence both gastric and intestinal motor function in humans and in an experimental setting [19–22].

We focused on determination of plasmatic concentration-time profile of memantine after single, repeated and additional administration. We found no correlation between EGG parameters and serum concentration or organ distribution of memantine. The high concentration in proximal jejunum is in good agreement with fast absorption after i.g. administration; the peak concentrations were reached within 75 to 150 min after administration in all cases. We observed little difference in the peak concentrations throughout the experiment, safe for the additional administration of 40 mg of memantine at the end of the experiment, where the concentrations more than doubled. This observation, nonetheless, corroborates the previously published data on linearity of memantine pharmacokinetics (in this case the standard dose of 20 mg with subsequent dose of 40 mg of memantine) [23]. The memantine levels were relatively stable after completion of absorption, which is well in line with the long elimination half-life described earlier [24]. This assumption is also supported by the strong distribution of memantine into peripheral organs and central nervous system, as seen in this work and in literature [25–27].

The ability to cross the blood brain barrier and to reach the site(s) of action are the mandatory prerequisites for successful anti-AD treatment [28,29]. Memantine is a cationic amphiphilic substance and as such should pass through biological membranes in its unionized form by passive diffusion [30]. Studies proving the blood-brain barrier permeation, active/facilitated transport or accumulation of memantine within the target brain structures are still rare. According to our results, the brain concentrations of memantine were more than twice as big as in plasma in all cases [25–27]. As we expected, the memantine concentration in various brain regions were comparable with peripheral fat concentration, indicating the vast distribution of the drug within tissues. These lipophilic depots are probably responsible for the long elimination half-life of memantine [26,27]. Difference in concentration of memantine in the pituitary gland compared to other brain regia suggests the involvement of blood-brain barrier. The direct connection of the pituitary gland and hippocampus might explain the relatively high concentration in the latter. This may also explain the pharmacodynamic effect of memantine via local MNDA receptors.

Memantine is primarily excreted unmetabolized via kidney, low concentration in the kidney tissue (compared to relatively higher concentration in urine) indicates active tubular secretion and low tubular reabsorption [31]. The small extent to which it is metabolized by CYP450 2B6 corresponds with the observed low concentration in liver tissue [32].

Memantine is believed to block the current flow through channels of NMDA glutamate receptor [33,34]. NMDA receptors are involved in the regulation of many gastric functions: **1)** hydrochloric acid production [35], **2)** glutamatergic, cholinergic and non-cholinergic pathways [36–38], **3)** ghrellin mediated processes [12], **4)** visceral pain [39], **5)** chronic *Helicobacter pylori* infection [40], **6)** gastric accommodation and emptying [41] and other. NMDA receptors also participate in the brain-gut axis regulation [42] and function of the lower oesophageal sphincter [43]. Thus, the complex action of this drug can **1)** explain the gastrointestinal dysmotility side effects of memantine and **2)** be possibly of use in treatment of dyspepsia and nausea [39,44]. Recently, data on possible memantine prophylaxis against soman poisoning were published. It appears memantine counteracts the poisoning symptoms via a combination of acetylcholinesterase-protection and NMDA receptor-blockade [45,46].

We used a single low dose of ketamine–another NMDA blocker–as introduction to anaesthesia, so possible gastric myoelectric effect of ketamine, might have influenced the basal EGG recording. The study of memantine effect itself remained however unaffected, because the mechanism of action of the two NMDA-receptor modulators is quite distinct, and because a blank reading was performed [47].

Surprisingly, we found no connection between EGG parameters and serum concentration or organ distribution of memantine. We hypothesize that memantine affects the myoelectric activity of the stomach directly by topical effect and by systemic plasmatic circulation.

## Conclusions

Both single and repeated doses of memantine increased DF. Severe gastric arrhythmia and long-lasting low power after repeated administration might explain possible gastric dysmotility side effects in the chronic use of memantine.

## Abbreviations

AD: Alzheimer's disease
DF: dominant frequency
EGG: electrogastrography
HPLC: high performance liquid chromatography
IQR: interquartile range
IS: internal standard
NMDA: N-methyl-D-aspartate type glutamate receptor
S.D.: Standard deviation
